# Treatment of Pathological Proximal Femur Fracture in a Child With a Unicameral Bone Cyst: A Case Report

**DOI:** 10.7759/cureus.67404

**Published:** 2024-08-21

**Authors:** Philip Lew Wei Sheng, Ahmad Fazly Abd Rasid, Kamal Jamil

**Affiliations:** 1 Department of Orthopedics and Traumatology, Faculty of Medicine, Hospital Canselor Tuanku Muhriz, Universiti Kebangsaan Malaysia, Kuala Lumpur, MYS; 2 Department of Pediatric Orthopedics, Faculty of Medicine, Universiti Kebangsaan Malaysia Medical Centre, Kuala Lumpur, MYS

**Keywords:** pediatric hip plate, synthetic bone graft, curretage, pathological femur fracture, unicameral bone cyst

## Abstract

Pathological proximal femur fractures secondary to unicameral bone cysts in the pediatric age group are uncommon. Despite the development of modern surgical treatments and implant choices, it is still debatable what the gold standard of treatment is for a unicameral bone cyst with pathological proximal femur fractures. We report a case of an eight-year-old child with a unicameral bone cyst and pathological fracture of the proximal right femur who was surgically treated with curettage, synthetic bone graft, and a pediatric hip plate as the choice of implant for osteosynthesis and stability.

## Introduction

The unicameral bone cyst (UBC), also known as a simple bone cyst, is a common and benign cystic lesion of the skeletal system [1]. Defined by Virchow in 1876, these are benign fluid-filled cavities often found in the metaphysis of long bones in children and adolescents, predominantly in the proximal humerus (55%-65%) and femur (25-30%) [2-4]. UBCs are mostly asymptomatic and are identified incidentally [5]. They account for 3% of all bone lesions in this age group, with about 75% of patients presenting with pathological fractures [2,4]. UBCs occur more frequently in boys than in girls, with a ratio of 2:1, and are most commonly seen in the first and second decades of life, with a reported peak between ages 3 and 14 years and an average age at diagnosis of 9 years [4,6]. The etiology of UBC is still debated, with trauma, inflammation, and venous obstruction of bones proposed as possible causes [5]. A more widely accepted theory suggests that a focal defect in metaphyseal remodeling impedes interstitial fluid drainage, leading to increased pressure from focal bone necrosis and the accumulation of fluid [2].

There are many treatment options for UBCs, including intralesional injections, resection with curettage and bone grafting, and local adjuvant treatments. A combination of techniques is also recommended in cases where the lesion is at high risk of refracture, especially in weight-bearing bones [7,8]. A combination of surgical treatments includes side plates and screws, pediatric hip plates, flexible intramedullary nailing, or dynamic hip screw to provide stability. According to current literature, recurrence occurs in up to 25% of cases and remains a concern despite modern surgical techniques [1].

## Case presentation

An eight-year-old boy with underlying G6PD deficiency presented to our center after slipping and falling at home. He landed on his right hip, experiencing immediate pain with a noticeable hip deformity. The child also had a history of falls while getting down from the bed two weeks before the current complaint. He landed feet first and noticed a dull, aching pain in his right hip. He had been limping since then but had improved over two weeks. He is the oldest of three siblings, and the other two are healthy. On examination of the right lower limb, the right hip was externally rotated, abducted, and shortened. There was tenderness on palpation over the greater trochanter, associated with swelling. Otherwise, the neurovascular examination of both lower limbs was unremarkable. Blood investigations did not detect any abnormalities (Table 1).

**Table 1:** Blood investigations during injury presentation.

Investigation	Result	Reference range	Unit
Hemoglobin	12.8	10.2-12.7	g/dL
White blood cell	20.6	6.5-13.0	x10^9^/L
Platelets	394	214-459	x10^9^/L
Sodium	138	136-146	mmol/L
Potassium	4.2	3.5-5.1	mmol/L
Urea	4.9	1.8-6.4	mmol/L
Creatinine	40.9	14-34	µmol/L
C-reactive protein	<0.1	<5	mg/L
Calcium	2.48	2.25-2.75	mmol/L
Magnesium	0.86	0.77-1.03	mmol/L
Phosphate	1.7	1.29-2.26	mmol/L
Albumin	43	35-52	g/L
Total protein	78	57-80	g/L
Alanine transaminase (ALT)	10	13-45	U/L
Aspartate aminotransferase (AST)	25	15-60	U/L
Total bilirubin	16.4	5-21	µmol/L

**Figure 1:**
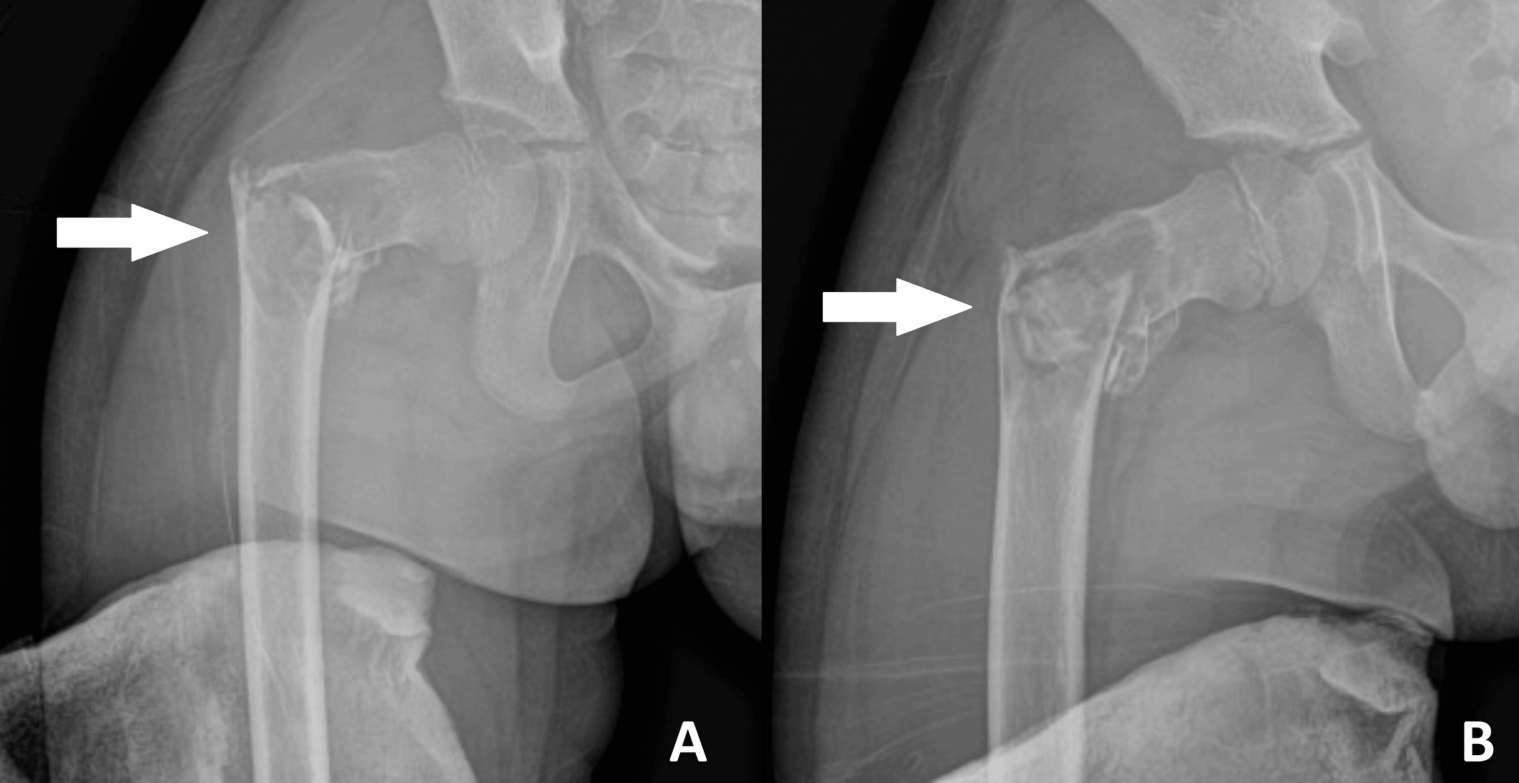
Plain radiographs of the right femur showing (A) AP and (B) lateral views at first presentation. There is a comminuted fracture of the right proximal femur at the intertrochanteric region with a centric expansile lesion and a thin lateral cortex, as indicated by the arrows. AP, anteroposterior

Radiographs of the right hip revealed a comminuted fracture of the intertrochanteric region of the femur, extending to the lesser trochanter, with a centric expansile lesion and a *fallen leaf* sign (Figure 1).

**Figure 2:**
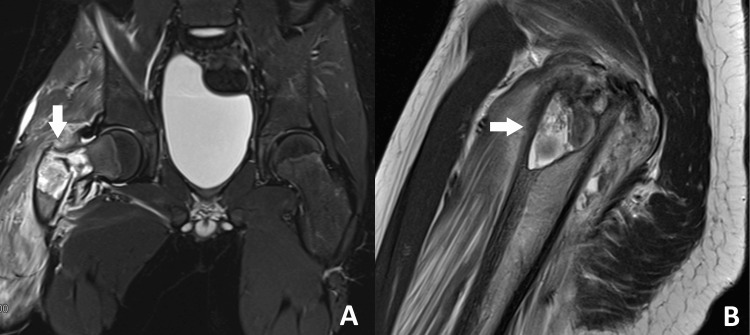
MRI of the proximal right femur showing (A) coronal and (B) sagittal views, demonstrating a hyperintense signal at the meta-diaphyseal region on T2, as indicated by the arrows. MRI, magnetic resonance imaging

The MRI of the right hip showed a well-defined intraosseous lesion at the right proximal metadiaphyseal region of the femur involving the neck, greater trochanter, and proximal shaft. The lesion demonstrated hyperintensities on T2 and hypointensities on T1, with a fluid-fluid level. There was a pathological fracture in the intertrochanteric region involving this lesion. The findings on the MRI were consistent with the diagnosis of a unicameral bone cyst, revealing a benign bone lesion at the proximal meta-diaphysis of the right femur with a pathological fracture (Figure 2).

**Figure 3:**
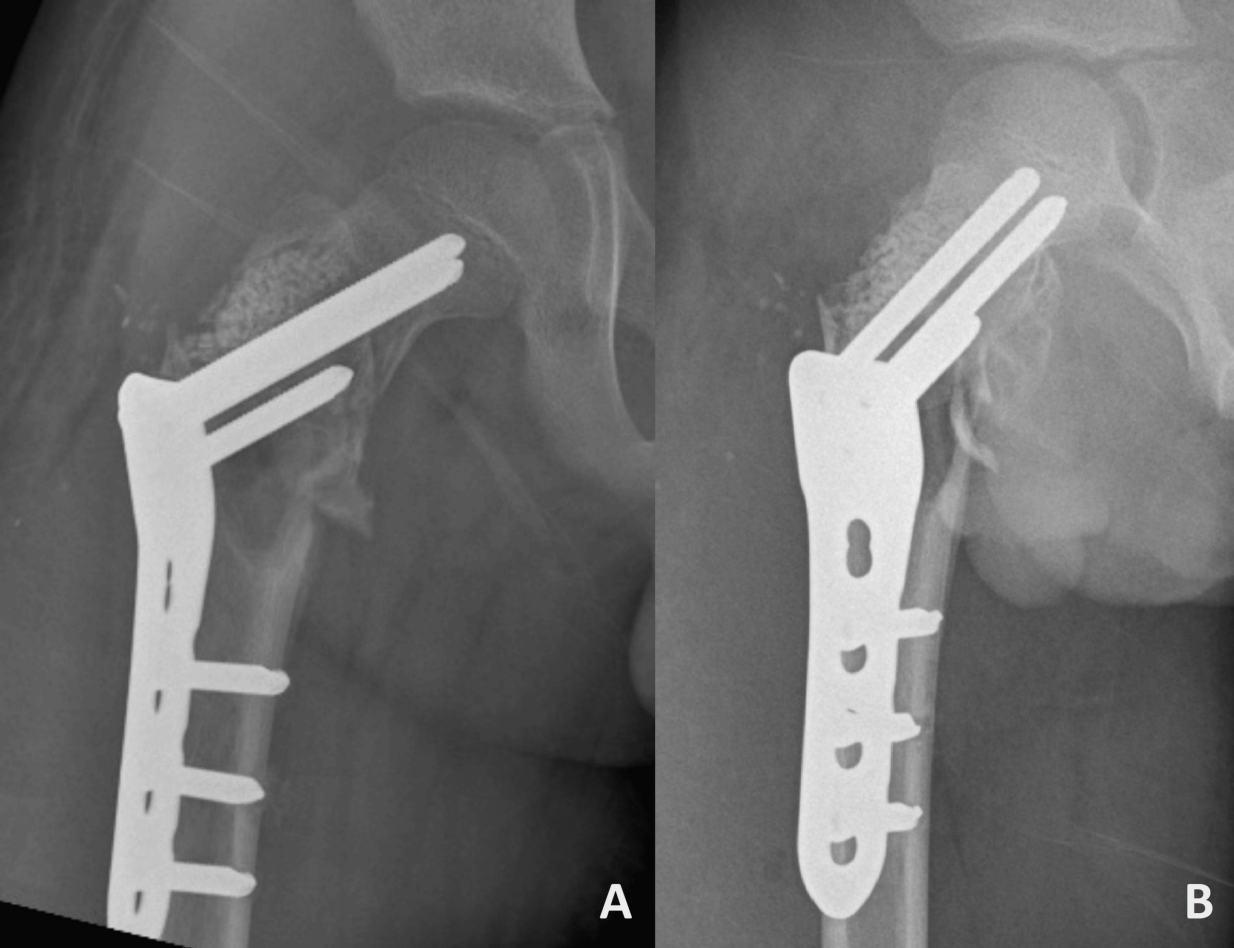
Initial postoperative radiographs showing (A) AP and (B) lateral views, demonstrating the implant in situ with restoration of anatomy. AP, anteroposterior

The patient underwent open reduction and internal fixation of the right proximal femur with a pediatric hip plate and curettage with a synthetic bone graft (Figure 3).

**Figure 4:**
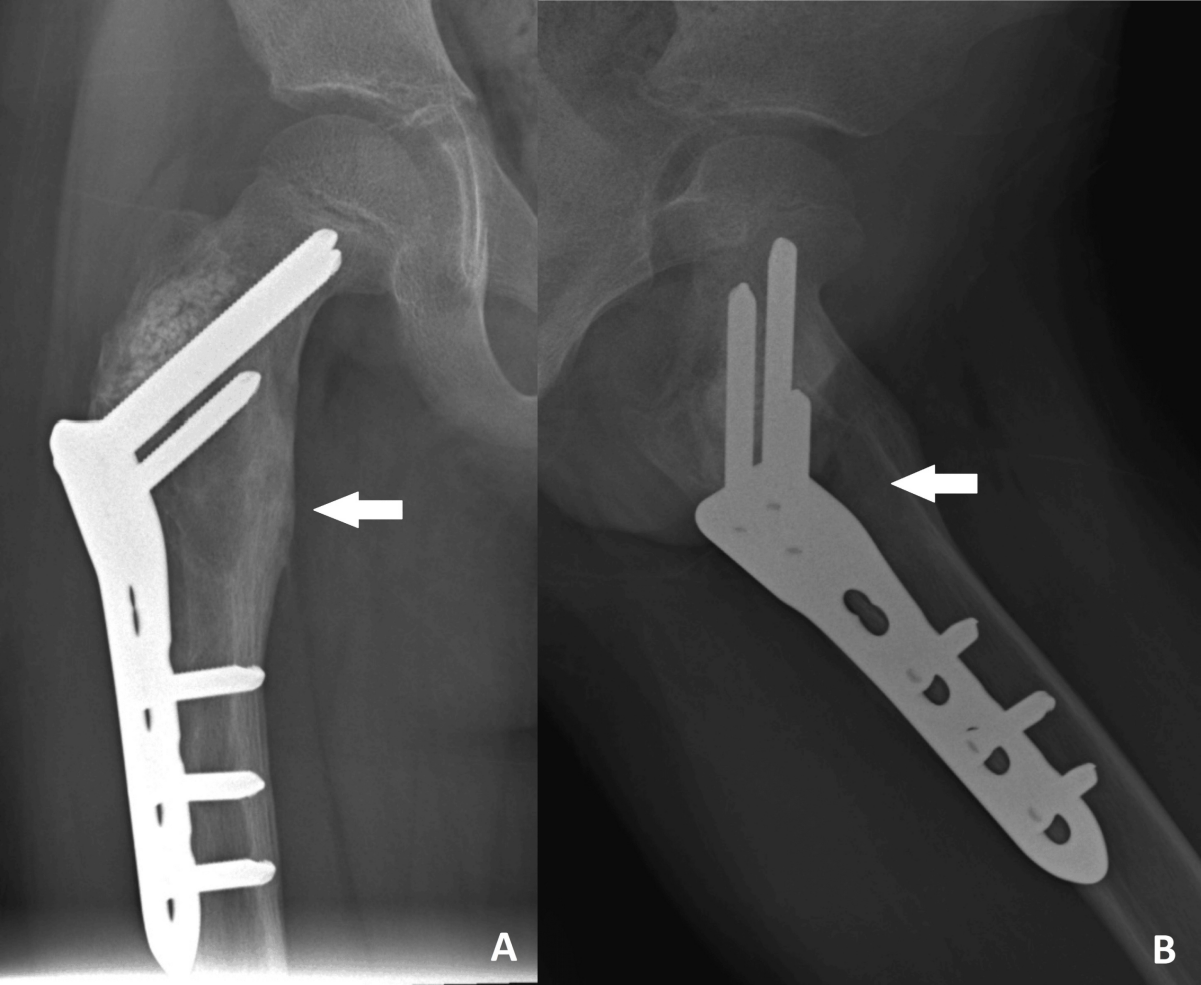
Plain radiographs of the right femur in (A) AP and (B) lateral views at the three-month follow-up showing progressive union of the fracture at the intertrochanteric region, with resolution of the cyst cavity, as indicated by the arrows. AP, anteroposterior

The histopathological findings revealed multiple fragments of lamellar bone tissue rimmed by osteoblasts and fibrous tissue, with areas covered by fibrin-like collagenous deposits and blood. There are also aggregates of histiocytes, along with focal reactive woven bone formation. No atypical cells or evidence of malignancy were seen. The results of the biopsy were consistent with a unicameral bone cyst. At the three-month follow-up, radiographs indicated a progressive union of the fracture with a resolution of the cyst cavity (Figure 4). The child was able to fully bear weight and had no complaints of pain.

## Discussion

The pathogenesis of the unicameral bone cyst remains unknown, despite etiological theories of inflammation, increased pressure within the cyst due to the accumulation of fluid from venous obstruction, and a higher concentration of lytic enzymes released by tissue lining the cyst wall, which promotes bone resorption [5]. The proposed treatments for these etiologies include intralesional steroid injections as described by Scaglietti, decompression techniques using intramedullary nails, cannulated screws, and Kirschner wires, and the classic curettage with bone grafting [6]. In pathological proximal femur fractures, a combined technique with internal fixation prevents complications of varus malunion, osteonecrosis of the femoral head, and growth arrest of the proximal femoral physis [6].

A pathological proximal femur fracture with a unicameral bone cyst warrants surgical intervention with fixation, as this is a weight-bearing bone, impairing the child’s ability to ambulate. There is a dilemma regarding the types of internal fixation to be applied to stabilize a pathological proximal femur fracture, so Dormans and Flynn proposed a classification based on the status of the physis and bone stock availability for internal fixation. This classification is helpful to assist and guide the surgeon in pre-operative planning and consideration of physeal integrity [9]. According to the classification, our patient is classified as type 1B, where the lateral buttress is lost but with sufficient bone beneath the physis for the purchase of a screw or a nail [9]. Internal fixation alone is insufficient, as it requires a combination of surgical procedures, including curettage with or without bone graft, and internal fixation, as described by Mavcic et al. [10]. This is true in our case, where we performed an open curettage, inserted a synthetic bone graft, and implemented the internal fixation. Canavese et al. explained that the procedure of curettage, has a mechanical effect whereby the cyst cavity is disrupted, allowing communication of the cavity with bone marrow, which leads to the activation of osteogenic potential for bone healing [11]. A bone graft substitute such as calcium sulfate and calcium phosphate has been shown to have osteoconductive properties with a prolonged resorptive period, which may lead to improved healing rates and interim bony stability, decreasing the need for revision surgeries in comparison with the use of allografts and autografts [12].

The use of flexible intramedullary nailing in proximal femur fractures for unicameral bone cysts has been reported by Murphy et al. that the outcomes of flexible intramedullary nailing in pediatric femoral fractures were excellent but with few complications, namely angulation of the fracture sites, especially the proximal and distal third of the femur [13]. Also, if applied to older children and children who are heavier, the outcomes are worse [13]. The localization of fractures in the basocervical and intertrochanteric regions of the proximal femur creates a difficult environment to provide stability with the use of flexible intramedullary nailing, leading to a higher complication rate and risk of revision surgery [14].

In cases where the lateral buttress of a proximal femur fracture is lost, a fixation with a lateral buttressing effect is used. Jamshidi et al. introduced the use of a proximal humeral locking plate in a group of pediatric patients, as the size matches the proximal femur. An additional strut graft was added to the neck of the femur, as their cases include Dormans Type IIB with insufficient bone stock proximal to the physis, which could also potentially reduce the risk of fracture and screw cutout. They evaluated 14 pediatric patients with a mean follow-up of 41.7 months and reported that none of the cases required re-operation, recurrence, fracture, or screw cutout. However, there were complications in three patients (21.4%) with infection, heterotopic ossification, and coxa vara [15]. A dynamic hip screw is another alternative, but Subramanyam et al. reported two cases of non-union of the neck of the femur; one was diagnosed with a unicameral bone cyst and treated initially with a dynamic hip screw. The patient required revision surgery given the reduced range of movement and shortening of the limb with radiological findings of non-union by open reduction, cancellous and fibular strut graft, valgus osteotomy, and a contoured 4.5 narrow dynamic compression plate. Their surgery improved the range of movements of the lower limb and achieved complete fracture union. They revised the surgery as the dynamic hip screw had failed osteosynthesis with non-union in the patient diagnosed with a unicameral bone cyst [16]. It is recommended by Baghdadi et al. to utilize fixation with lateral buttress for all cysts around the hip. In this review, a screw and side plate are used, but depending on the location and extent of the physeal injury, there is a possibility for angular deformity and growth disturbance [1]. Finally, Tomaszewski et al. applied a pediatric hip plate for the surgical treatment of 15 patients with unicameral bone cysts. It was observed that 13.3%, or 2 of the 15 patients, had recurrence, which required additional curettage and bone grafting. They suggested in the management of unicameral bone cysts to remove the lesion completely, fill the cavity with osteoconductive material, and stabilize the proximal femur with plate fixation, as 13 of the 15 (86.7%) patients achieved consolidation [17].

In our case, we opted for pediatric hip plate fixation due to its numerous advantages. Firstly, this specific variant of the plate was readily available at our center, which ensures the surgery can proceed without any unnecessary delay and provides early surgical treatment for pain reduction, to obtain a biopsy for histological examination, and for early mobilization [14]. Additionally, the anatomical contour of the plate is well-fitted to the proximal femur bone of the child, ensuring better plate-to-bone contact for effective stabilization and long-lasting anatomical correction [14]. Besides, the low plate profile is thin and minimally intrusive on the delicate soft tissue of the child with no severe local irritations, and the availability of a plate design with three holes facilitates insertion and fixation with less soft tissue and muscle stripping [18]. On top of that, the size of the plate and screws is dependent on the patient’s age and weight; they are specifically suited for pediatric patients, accommodating their natural immature skeletal structure [18].

## Conclusions

Pathological fractures in the proximal femur resulting from unicameral bone cysts in the pediatric population require surgical intervention with internal fixation, as this will contribute to postoperative rehabilitation and an early return to daily activities. It is crucial to understand the location of the lesion, bone stock availability, and lateral buttress patency, as these parameters will guide the surgeon in the course of pre-operative planning and surgical implant selection. In this instance, the decision to use a pediatric hip plate had good outcomes, with radiological evidence showing progressive fracture union and a resolved cyst cavity. Most importantly, the patient was able to bear weight fully and return to daily activities as a child.

## References

[1] Baghdadi S, Arkader A (2021). Unicameral bone cysts: treatment rationale and approach. J Pediatr Orthopaedic Soc North Am.

[2] Kim MC, Joo SD, Jung ST (2018). The role of fractures on pathologic bone in healing of proximal humerus unicameral bone cysts. J Orthop Surg (Hong Kong).

[3] Erol B, Onay T, Çalışkan E, Aydemir AN, Topkar OM (2015). Treatment of pathological fractures due to simple bone cysts by extended curettage grafting and intramedullary decompression. Acta Orthop Traumatol Turc.

[4] Pogorelić Z, Furlan D, Biocić M, Mestrović J, Jurić I, Todorić D (2010). Titanium intramedullary nailing for treatment of simple bone cysts of the long bones in children. Scott Med J.

[5] Bukva B, Vrgoč G, Abramović D, Dučić S, Brkić I, Čengić T (2019). Treatment of unicameral bone cysts in children: a comparative study. Acta Clin Croat.

[6] Pretell-Mazzini J, Murphy RF, Kushare I, Dormans JP (2014). Unicameral bone cysts: general characteristics and management controversies. J Am Acad Orthop Surg.

[7] Noordin S, Allana S, Umer M, Jamil M, Hilal K, Uddin N (2018). Unicameral bone cysts: current concepts. Ann Med Surg (Lond).

[8] Döring K, Sturz GD, Hobusch G, Puchner S, Windhager R, Chiari C (2023). Open surgical treatment of unicameral bone cysts: a retrospective data analysis. Wien Klin Wochenschr.

[9] Cruz Jr AI, Lindskog D (2011). Pathologic fractures through benign bone lesions in children and adolescents. Curr Orthop Prac.

[10] Mavčič B, Saraph V, Gilg MM, Bergovec M, Brecelj J, Leithner A (2019). Comparison of three surgical treatment options for unicameral bone cysts in humerus. J Pediatr Orthop B.

[11] Canavese F, Wright JG, Cole WG, Hopyan S (2011). Unicameral bone cysts: comparison of percutaneous curettage, steroid, and autologous bone marrow injections. J Pediatr Orthop.

[12] Nunziato C, Williams J, Williams R (2021). Synthetic bone graft substitute for treatment of unicameral bone cysts. J Pediatr Orthop.

[13] Murphy JS, Koehler R, Johnson M, Hosseinzadeh P (2021). Flexible intramedullary nailing of pediatric femoral fractures. JBJS Essent Surg Tech.

[14] Patrikov K, Georgiev H, Kehayov R (2022). Pathological fractures of the proximal femur in children and adolescents treated with LCP paediatric hip plate. Acta Chirurgiae Orthopaedicae et Traumatologiae Čechoslovaca.

[15] Jamshidi K, Mirkazemi M, Izanloo A, Mirzaei A (2018). Locking plate and fibular strut-graft augmentation in the reconstruction of unicameral bone cyst of proximal femur in the paediatric population. Int Orthop.

[16] Subramanyam KN, Mundargi AV, Reddy PS, Umerjikar S (2018). Pathological neck of femur fracture with failed osteosynthesis in adolescent: A report of two cases. J Orthop Case Rep.

[17] Tomaszewski R, Rutz E, Mayr J, Dajka J (2022). Surgical treatment of benign lesions and pathologic fractures of the proximal femur in children. Arch Orthop Trauma Surg.

[18] Joeris A, Audigé L, Ziebarth K, Slongo T (2012). The locking compression paediatric hip plate™: technical guide and critical analysis. Int Orthop.

